# Analysis of the Ability of Capsaicin to Modulate the Human Gut Microbiota In Vitro

**DOI:** 10.3390/nu14061283

**Published:** 2022-03-18

**Authors:** Karley K. Mahalak, Jamshed Bobokalonov, Jenni Firrman, Russell Williams, Bradley Evans, Brian Fanelli, Jason W. Soares, Masuko Kobori, LinShu Liu

**Affiliations:** 1 Dairy and Functional Foods Research Unit, Eastern Regional Research Center, Agricultural Research Service, United States Department of Agriculture, 600E Mermaid Lane, Montgomery, PA 19038, USA; jamshedbt@gmail.com (J.B.); jenni.firrman@usda.gov (J.F.); linshu.liu@usda.gov (L.L.); 2 Proteomics and Mass Spectrometry Facility, Donald Danforth Plant Science Center, 975 North Warson Road, St. Louis, MO 63132, USA; rwilliams@danforthcenter.org (R.W.); bevans@danforthcenter.org (B.E.); 3 CosmosID Inc., 1600 East Gude Drive, Rockville, MD 20850, USA; brian.fanelli@cosmosid.com; 4 Soldier Effectiveness Directorate, US Army Combat Capabilities Development Command Soldier Center, Middlesex, MA 01760, USA; jason.w.soares.civ@army.mil; 5 Food Research Institute, National Agriculture and Food Research Organization, Tsukuba 305-8642, Japan; kobori@affrc.go.jp

**Keywords:** capsaicin, gut microbiota, short-chain fatty acids (SCFAs), untargeted metabolomics

## Abstract

Previous studies on capsaicin, the bioactive compound in chili peppers, have shown that it may have a beneficial effect in vivo when part of a regular diet. These positive health benefits, including an anti-inflammatory potential and protective effects against obesity, are often attributed to the gut microbial community response to capsaicin. However, there is no consensus on the mechanism behind the protective effect of capsaicin. In this study, we used an in vitro model of the human gut microbiota to determine how regular consumption of capsaicin impacts the gut microbiota. Using a combination of NextGen sequencing and metabolomics, we found that regular capsaicin treatment changed the structure of the gut microbial community by increasing diversity and certain SCFA abundances, particularly butanoic acid. Through this study, we determined that the addition of capsaicin to the in vitro cultures of the human gut microbiome resulted in increased diversity of the microbial community and an increase in butanoic acid. These changes may be responsible for the health benefits associated with CAP consumption.

## 1. Introduction

Capsaicin (CAP) is the bioactive compound found in red pepper that provides the associated pungent flavor. CAP has been historically used to mask the poor taste of deteriorated food, add flavor to enhance cuisine, and for medicinal purposes [1,2,3]. In humans and mice, a regular diet containing CAP is associated with many positive health effects, including lowered cholesterol and obesity, as well as having antioxidant, anti-inflammatory, and anti-hypertensive effects [4,5,6,7,8,9,10,11,12]. Recently, there have been more efforts to determine the mechanisms behind these positive health impacts of dietary CAP. The anti-inflammatory and pain-relieving abilities of CAP have been associated with its action as an agonist for the transient receptor potential cation channel subfamily V member 1 (TRPV1) [13,14,15,16]. Additionally, the positive impacts of CAP on dietary health may be due to the interaction between CAP and the gut microbiota [17]. 

The gut microbiota is an impactful mediator of health and human disease [18,19]. Dysbiosis of the gut microbiota, often correlated with a lack of diversity that may occur for many reasons, which include increasing age and a western diet, is a contributor to adverse health conditions such as obesity, diabetes, and irritable bowel disease [20,21,22,23]. In vivo studies have shown that CAP can alter the gut microbial population at the genus level in mice and humans. In two in vivo mouse studies, dietary CAP reduced weight gain and food intake and increased population numbers of key gut microbial genera, such as *Bacteroides*, *Akkermansia*, and *Prevotella,* while reducing population numbers of *Escherichia* and *Sutterella* [24,25]. These changes in gut microbial composition are likely to account for many of the positive health changes associated with CAP treatment. Another in vivo study revealed a significant change in gut microbial community diversity with dietary CAP, including an increase in *Lachnospiraceae* and *Ruminococcaceae*, species that produce short-chain fatty acids (SCFAs), especially butanoic acid [26]. Conversely, a further in vivo mouse study found that CAP consumption decreased *Bacteroides* and *Parabacteroides,* though CAP was associated with an increase in SCFA production [27]. These changes in the bacterial population are reflected in changes in SCFA production and bile acid metabolism with dietary CAP as well [24,28]. It has been speculated that these SCFA changes that occur due to shifts in the gut microbial community are the cause of the positive health effects associated with CAP, including the promotion of glucose homeostasis. Due to the rising instances of obesity, diabetes, and irritable bowel disease, it is imperative to research the effect of CAP on the gut microbiota for the possibility of its use as a preventative measure to the aforementioned disease.

Previous efforts to elucidate mechanistic effects of CAP and how they impact the gut microbiota have largely been performed in vivo [29,30]. In this study, we used a simplified in vitro approach to clarify the impact of CAP on the healthy, human colonic gut microbial community without compounding factors of the host organism. This method allowed us to provide a more in-depth understanding of the microbial interactions to delineate the role of the host and microbiome interactions as a function of CAP supplementation. To do so, in vitro human gut microbial communities were established and treated with CAP for 2 weeks; the communities were then analyzed using metagenomics and metabolomics methods.

## 2. Materials and Methods

### 2.1. Materials

The starting homogenate of each individual human fecal sample (Microbiome Health Research Institute, Boston, MA, USA) was harvested from a single American, randomly selected as described previously [31,32], for a total of 2 individual human fecal samples. The pancreatic juice (PJ) and defined medium (DM) were prepared as described previously [32]. Capsaicin (CAP) was purchased from Sigma Aldrich (St. Louis, MO, USA). 

### 2.2. Preparation of Capsaicin

Capsaicin was dissolved in a concentration of 300 mg in 100 mL of pure PEG (200 g/mL molecular weight). A total of 25 mL of this solution once a day for day 1 and 7.5 mL twice a day from day 2 to 14 were injected into two bioreactors. As a control, the same amount of pure PEG for two and water for one bioreactor (25 mL once a day for day one and 7.5 mL twice a day from day 2 to 14) was injected. 

### 2.3. Human Gut Microbial Community In Vitro Experiment

BioFlow320 bioreactors (Eppendorf) were used to perform the in vitro experiments. Each bioreactor experiment was started with inoculum from a different individual, for a total of 2 different inoculums. For this study, they are differentiated using C1 and C2. C1 was used to inoculate 4 BioFlow320 bioreactors, 1 water control, 1 PEG control, and 2 experimental reactors. C2 was used to inoculate 5 BioFlow320 bioreactors, 1 water control, 2 PEG controls, and 2 experimental reactors. Each bioreactor was set up to mimic a single adult large colon under the following conditions, as described previously: a pH of 7.0 ± 0.1 and a temperature of 37 °C, and anaerobiosis were maintained with a N_2_ flow [33]. DM and PJ were fed to each community at 8-h intervals throughout the experiment as described previously [33].

For C1 and C2, after inoculation, the community was maintained for 2w to allow for the stabilization of the bacterial community. The community was maintained with 3× a day feeding cycles as described previously [32]. After stabilization, CAP was added to each experimental bioreactor for 14 days. Samples were taken throughout this study, approximately every second day, just before new DM was added to the reactor. Samples for bacterial analysis were pelleted by centrifugation and stored at −80 °C until they were analyzed. Samples for metabolic analysis were filter-sterilized (0.2 µm filter) and stored at −80 °C until they were analyzed. These samples were categorized into 3 main phases: stabilization (at the end of the 2-week stabilization period), CAP Start (the first 3 time-points immediately following the addition of CAP), and CAP end (the last 3 time-points of the treatment period).

### 2.4. Quantification of Short Chain Fatty Acids by GC-MS

The samples were collected in 5 mL centrifuge tubes and stored at −80 °C. The set of samples was thawed just before analysis. An aliquot of 250 μL of the sample was added with 12.5 µL of Internal Standard (1600 mg/L), 62.5 µL of 49% Sulfuric acid, 50 mg of Sodium chloride, and 1 mL Diethyl ether in 2 mL centrifuged tubes. Finally, tubes were shaken in a HulaMixer (Lifetechnologies, ThermoFisher Scientific, Waltham, MA, USA) for 3 min and centrifuged at 664× *g* for 3 min, and the organic solvent layer was transferred to a vial, loaded to GC autosampler, and analyzed using GC-MS. Each sample was prepared and processed in triplicate.

The SCFAs in the DM were analyzed using a GC/MS Shimadzu QP2010 Ultra (Shimadzu, Columbia, MD, USA) equipped with a Stabilwax-DA column, 30 m, 0.25 mm ID, 0.25 μm (Restek Corporation, Bellefonte, PA, USA). The following instrument settings were used: an initial temperature of 125 °C was held for 1 min; then, it was increased to 170 °C at 30 °C/min, then to 181.5 °C at 20 °C/min and held 0.5 min; finally, it grew up to 220 °C at 50 °C/min for 2 min. A total of 1 µL of the sample was injected in a 1:20 split mode at 260 °C; the interface and ion source temperatures were 280 °C and 220 °C, respectively. A standard stock solution containing acetic acid, propionic acid, isobutyric acid, butyric acid, 2-methylbutyric acid, isovaleric acid, valeric acid, 2-methylvaleric acid, 3-methylvaleric acid, 4-methylvaleric acid, hexanoic acid, and heptanoic acid all in concentrations of 5 mg/mL was prepared by dissolving the analytical standards in 0.1 M NaOH. The mixed standard working solutions were prepared using serial dilution in a concentration range of 0.0125–5 mg/mL. The internal standard stock solution was prepared by dissolving 2-methylhexanoic acid (1.6 mg/mL) in 0.1 M NaOH. All standard stock solutions were stored at −80 °C. The data were acquired in full scan mode with a mass range of *m*/*z* 25–375. The SCFA data used for this analysis can be found in Supplemental Table S1, in the supplemental data excel file. 

### 2.5. Untargeted Metabolomics Using LC-MS

Samples were filter sterilized using 0.2 µm filters prior to submission for LC-MS. Sample injections (2 µL) were analyzed using a 0.5 × 150 mm C18 column (Targa, C18, 3 µm, Higgins Analytical, Inc., Mountain View, CA, USA) eluted with water and acetonitrile, each containing 0.1% formic acid. The gradient profile used for elution was 2% to 100% acetonitrile from 3 to 13 min and a return from 100% to 2% from 16 to 19 min, followed by 11 min at 2% acetonitrile to allow the column to re-equilibrate. MS data were recorded in positive and negative mode using polarity switching using a Q-Exactive mass spectrometer set to a resolution setting of 70,000 and scan range extending from 100 to 1200 Da. Positive and negative MSMS data were acquired separately using randomly pooled samples covering the sample set with a scan range extending from 150 to 1200 Da. All data were acquired in triplicate.

Data were analyzed with Compound Discoverer 3.0 (ThermoFisher Scientific, Waltham, MA, USA) using a modified untargeted metabolomics workflow. The resulting compound list was filtered to remove entries identified as PEG, phthalate, glycol, fluorine-containing, and background compounds (compounds also found in solvent blanks). Any compounds that were not assigned a formula or a name were removed, as were those that did not have at least one “Annotation Source” match. Significant organism and metabolite associations were identified using the MaAsLin2 tool from the Huttenhower lab, based on the genus and species matrices from CosmosID, as well as untargeted metabolite abundances. The relative abundance of bacterial results was used, using the MaAsLin2 package’s default parameters [34]. Significance data for these results were determined based on the q value to account for false positives. The data files used for this analysis can be found in Supplemental Tables S2 and S3 in the supplemental data excel file. 

### 2.6. Shotgun Sequencing

DNA extractions were performed from pelleted bacterial samples using the DNEasy Powersoil Kit (Qiagen, Germantown, MD, USA). Unassembled sequencing reads were directly analyzed by the CosmosID bioinformatics platform (CosmosID Inc., Rockville, MD, USA) described elsewhere [35,36,37,38] for multi-kingdom microbiome analysis and the quantification of organisms’ relative abundance. Briefly, the system utilizes curated genome databases and a high-performance data-mining algorithm that rapidly disambiguates hundreds of millions of metagenomic sequence reads into the discrete microorganisms, engendering the particular sequences. The OTU file for this analysis can be found in Supplemental Table S4, in the supplemental data file. 

### 2.7. Bioinformatics and Statistical Analysis

**Figure 1:**
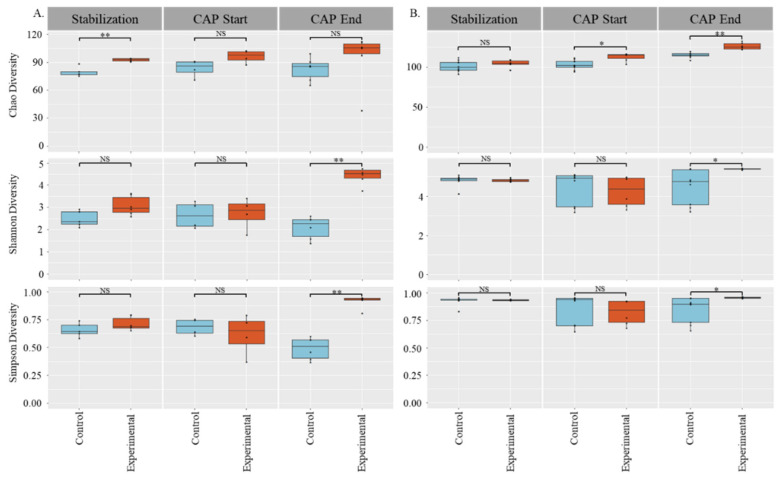
Alpha Diversity of Stabilization, CAP start, and CAP end phases: (**A**) C1, (**B**) C2, top: Chao diversity; middle: Shannon’s diversity; bottom: Simpson’s diversity. Significance determined by Wilcoxon Rank-Sum tests: * = *p* < 0.05, ** = *p* < 0.01.

Alpha diversity boxplots were calculated from the species-level filtered abundance score matrices from the CosmosID taxonomic analysis. Chao, Simpson, and Shannon alpha diversity metrics were calculated in R using the R package Vegan [39]. Wilcoxon Rank-Sum tests were performed between groups using the R package ggsignif [40]. Boxplots with overlaid significance levels were generated using the R package ggplot2 [41]. An analysis of the water control compared with the PEG control revealed no difference between the two in terms of community diversity (data not shown) and have therefore been combined for Figure 1. The stabilized communities from each host were significantly different from each other (*p* < 0.05); consequently, C1 and C2 were not combined for analysis.

Beta Diversity Principal Coordinate Analyses were calculated from the species-level filtered relative abundance matrices from the CosmosID taxonomic analysis. Bray–Curtis and Jaccard diversities were calculated in R using the R package Vegan with the functions vegdist, and PCoA tables were generated using Vegan’s function pcoa. PERMANOVA tests for each distance matrix were generated using Vegan’s function adonis2 [39]. PERMANOVA between each pair of groups was generated using pairwise.adonis2 from the pairwiseAdonis library [42]. Plots were visualized using the R package ggpubr [43].

## 3. Results and Discussion

### 3.1. Gut Microbial Diversity Increases with CAP Treatment

For this study, the effect of CAP on the gut microbial community from two independent donors was evaluated (C1 and C2). For each donor, there was a water control group to account for any changes PEG caused, a PEG control group, and an experimental group that was treated with CAP dissolved in PEG. The effect of CAP on the gut microbiota was analyzed at the beginning of CAP addition (CAP Start) and at the end of the 14 days of CAP treatment (CAP End). To understand how CAP impacts the gut microbial community structure in vitro, we performed shotgun sequencing and used both alpha and beta diversity measures to analyze the results. 

We used 3 different measurements of alpha diversity to obtain a clear picture of the impact of capsaicin on the colonic gut microbiota. Chao diversity is based only on species abundance, the Shannon’s diversity index is a measure based on species richness and evenness, and the Simpson’s diversity index is based on present taxa and abundance. For C1 and C2, there was a significant increase in community diversity at the end of CAP treatment when compared with control for both the Shannon’s diversity index and Simpson’s diversity index measures (*p* < 0.05). However, the two host communities had different responses with respect to Chao diversity. For C1, there was a difference in diversity during the stabilization phase (pre-addition of CAP) that normalized after CAP supplementation, showing an increase in Chao diversity in both the control and CAP-treated microbial communities. In C2, there was no difference between the control and experimental groups during the stabilization phase, but once CAP treatment began, the microbiome community increased in diversity with time (*p* < 0.05). 

**Figure 2:**
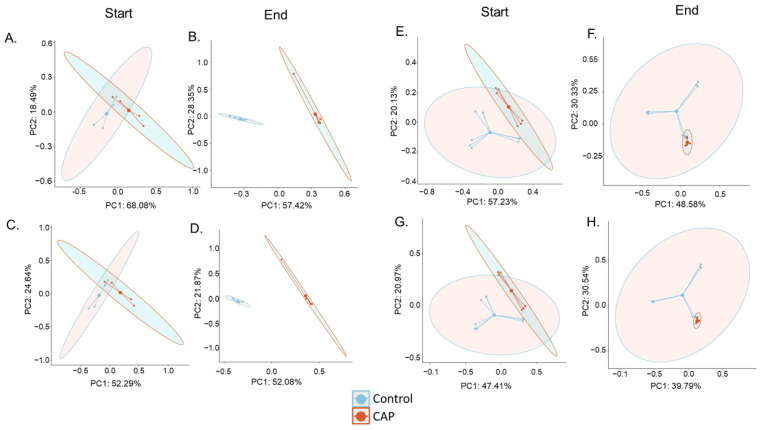
Beta Diversity of the CAP Start and CAP End phases. (**A**) C1 Bray–Curtis PCoA Start of Treatment (NS), (**B**) C1 Bray–Curtis PCoA End of Treatment (*p* < 0.001), (**C**) C1 Jaccard Index PCoA Start of Treatment (NS), (**D**) C1 Jaccard Index PCoA End of Treatment (*p* < 0.01), (**E**) C2 Bray–Curtis PCoA Start of Treatment (*p* < 0.05), (**F**) C2 Bray–Curtis PCoA End of Treatment (*p* < 0.05), (**G**) C2 Jaccard Index PCoA Start of Treatment (*p* < 0.05), and (**H**) C2 Jaccard Index PCoA End of Treatment (*p* < 0.001). Significance was determined using PERMANOVA.

Two measures of beta diversity were determined: the Jaccard index PCoA, which is determined based on the presence or absence of species, and the Bray–Curtis PCoA, which is determined based on the abundance of present species. The C1 gut microbial community, seen in Figure 2A–D, exhibited a significant (*p* < 0.01) shift in beta diversity in both the Bray–Curtis and Jaccard indices by the end of CAP treatment, where there was not a significant difference in the beta diversity of that community at the CAP Start or during the stabilization phase of the experiment. The C2 gut microbiota, shown in Figure 2E–H, also had a shift in the beta diversity of the community, but it is worth noting that the experimental groups were already significantly different from the control groups at the start of treatment (*p* < 0.05). However, in the C2 community, the Bray–Curtis PCoA plot showed no significant difference between the control and CAP-treated groups during the stabilization phase of the experiment, indicating that CAP treatment did alter the community structure immediately in abundance measurements alone. Conversely, the Jaccard index measurement showed that the control and experimental groups were significantly different (*p* < 0.05) during the stabilization phase (data not shown). 

Both CAP-treated communities (C1 and C2) exhibited significant changes in community diversity using multiple alpha and beta diversity measures. This is consistent with previous in vivo reports that found that CAP consumption in mice and humans changes the gut microbial community [24,26,28]. The corroboration of our findings with those of other researchers illustrates the ability of this in vitro model to mimic the in vivo gut microbial community response to CAP. Observed shifts in the community in this study indicate that CAP increases diversity in the gut microbial community. An increase in gut microbial diversity has been associated with better health, whereas a decrease in gut microbial diversity is associated with some illnesses, particularly in regards to type 2 diabetic individuals [44,45]. Thus, an increase in the gut microbial diversity illustrated here is indicative of the ability of CAP to modulate the gut microbiota beneficially. However, it is worth noting that C1 and C2 exhibited slightly different responses to CAP treatment. This is likely due to C1 and C2 having different starting communities. This difference in the gut microbial community response to CAP has been documented before in humans [30].

### 3.2. Relative Abundance of the Microbial Community Shifts in Unexpected Ways with CAP

**Figure 3:**
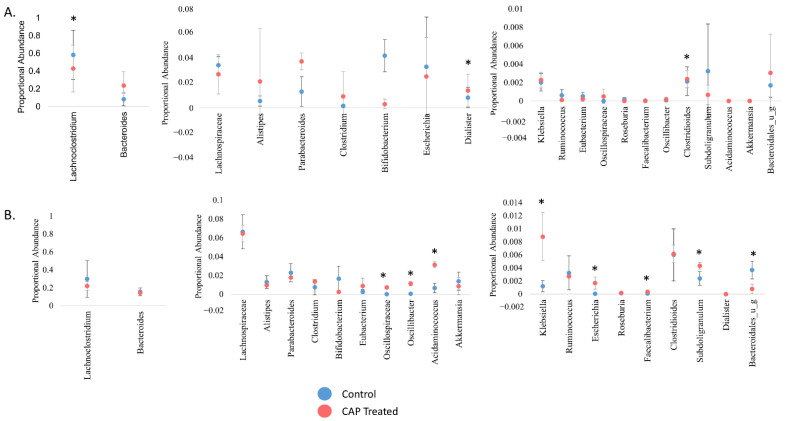
Proportional abundance of key gut microbiota, genus level. The proportional abundance in this instance references the abundance of a genus in comparison to others present at that time point. (**A**) C1, (**B**) C2. Significance was determined using a student’s t-test. * = *p* < 0.05.

To look more closely at the C1 and C2 bacterial communities with CAP treatment, the shotgun sequencing data was also used to determine the proportional abundance of genera that have previously been associated with consumption of CAP, as well as those that did exhibit a significant change in abundance in the current study (Figure 3). A comparative analysis was also performed to determine the differences between the C1 and C2 communities in response to CAP treatment. 

Interestingly, none of the genera that have been previously identified as affected by dietary CAP shifted significantly from control in the current study [24,26,28]. This discrepancy may be due to our use of human inoculum as opposed to the mouse studies done previously. However, there was a marked difference in which genera had a significant shift in abundance with CAP treatment between C1 and C2. For example, extrapolating from previous mouse studies, we expected that *Bacteroides* abundance would increase with CAP treatment; this did occur in C1, but not to a statistically significant extent, and it did not occur at all in C2. The relative abundance of *Akkermansia* was also expected to increase, which did not occur, possibly due to the lack of a solid mucosal surface in our model. In C1 (Figure 3A), *Lachnoclostridium* decreased in abundance with CAP treatment, whereas *Dialister* and *Clostridioides* significantly increased in abundance with treatment (*p* < 0.05). In C2 (Figure 3B), *Oscillospiraceae*, *Oscillibacter*, *Acidaminococcus*, *Escherichia*, *Faecalibacterium*, and *Subdoligranulum* increased with CAP treatment, whereas *Bacteroidales* decreased with treatment (*p* < 0.05). A gut microbial community with low levels of *Faecalibacterium* is associated with negative health effects such as obesity and inflammatory bowel disease [17,46,47]. Some members of *Escherichia* have demonstrated beneficial effects on the human gut microbiome as well, producing SCFAs that are beneficial to human health, especially including butyric acid [48]. The findings in this study that *Faecalibacterium* and *Escherichia* genera increased in abundance in C2 due to CAP supports previous work that indicated that CAP is beneficial to human health through changes to the gut microbiota. With the exclusion of *Escherichia* and *Bacteroidales,* all significantly altered genera in C1 and C2 are members of the Firmicutes phylum.

When this proportional abundance data is compared with Chao diversity, the difference in the response between C1 and C2 is not surprising. In Figure 1, the C1 community did not have a significant change in the abundance of species. However, C2 had a significant increase in species abundance (*p* < 0.01) at the end of CAP treatment. These results illustrate that CAP has a donor-dependent effect. This finding is further supposed by previous work that indicated that the effect of CAP is dependent on different gut enterotypes in humans, as well as findings that the effect of CAP on the gut microbiota was found to be sex-dependent in mice [29,30].

### 3.3. CAP Treatment Changes the Abundance of Key SCFAs

**Figure 4:**
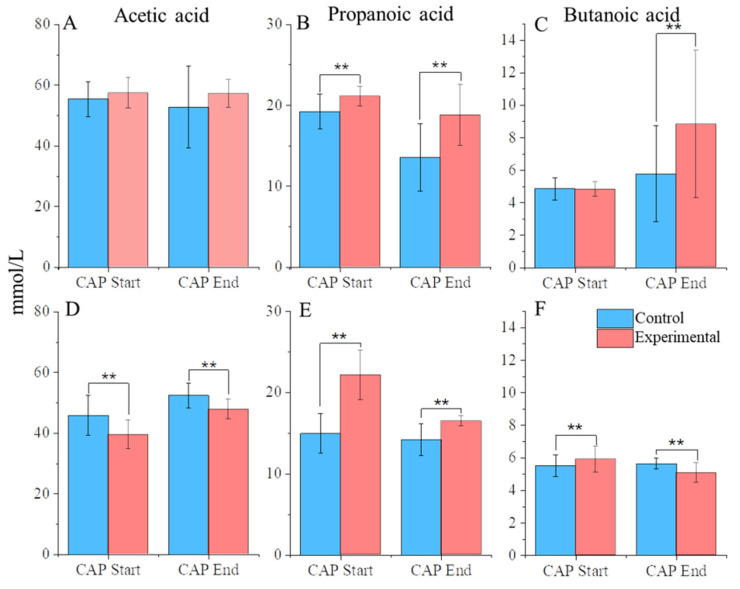
SCFA abundance. (**A**) C1 acetic acid, (**B**) C1 propanoic acid, (**C**) C1 butanoic acid, (**D**) C2 acetic acid, (**E**) C2 propanoic acid, (**F**) and C2 butanoic acid. ** = *p* < 0.01.

In vivo studies have shown that dietary CAP can change the abundance of short-chain fatty acids (SCFAs) [24,26]. It has been speculated that this shift in SCFA production is the reason for the positive health impacts of dietary CAP. It is well established that SCFAs are a major component in the regulation of gut health and overall health [49]. Butanoic acid is a key fuel source to intestinal epithelial cells, improving bacterial adhesion and the integrity of tight-junctions [50,51]. Propanoic acid is a key player in the regulation of appetite and, through that mechanism, the maintenance of body weight, and it has a beneficial effect on β-cell function and glucose homeostasis [52,53]. To determine whether shifts in SCFA production are observed in vitro, we performed GC-MS/MS to determine the abundance of key SCFAs, shown in Figure 4. As expected, the 3 most abundant SCFAs were acetic acid, propanoic acid, and butanoic acid [31,32,33,54]. 

In C1, acetic acid maintained a stable, high level of abundance throughout the experiment regardless of CAP treatment. Propanoic acid levels, however, were significantly increased between CAP-treated communities compared with the control, even during the stabilization phase, indicating that any differences between the two could be attributed to different starting amounts. Butanoic acid levels, however, significantly increased at the end of CAP treatment compared with control (*p* < 0.01).

In C2, there was a drop in the abundance of acetic acid (*p* < 0.01). corresponding with the beginning of CAP treatment. Propanoic acid levels significantly increased between the CAP-treated communities compared with the control from the stabilization phase, similarly to C1. Butanoic acid, however, did show a differential response between CAP-treated communities and the control, although the response was dependent on the experimental phase. At the CAP start, the CAP-treated group showed a significant increase in butanoic acid compared with the control, but this trend was reversed by the end of the treatment period (*p* < 0.01) to have a decrease in the concentration compared with the control. 

The more interesting effect lay in butanoic acid production between C1 and C2. In C1, we observed a steady state of butanoic acid production between the control and experimental groups through the CAP Start. However, we saw a notable increase in butanoic acid at the end of CAP treatment. Given what is known about the positive impact of butanoic acid on health, this indicates that CAP altered the gut microbiota of C1 in a way that was beneficial to the bacterial community. Conversely, in C2, we observed no increase, and in fact, we observed a slight decrease in the abundance of butanoic acid with the addition of CAP. This difference between C1 and C2 is particularly interesting, as a study published in 2020 showed no increase in butanoic acid, with an increase in acetic acid and propanoic acid with CAP treatment in mice [24]. However, a previous mouse study published in 2017 did illustrate a significant increase in butanoic acid with CAP treatment [26]. Previous work has suggested that observed differences in the health response of individuals may be due to different gut enterotypes [30]. This difference in the fundamental structure of the starting gut microbiota is therefore the likely explanation for the difference in response we observed between the C1 and C2 communities. 

### 3.4. Untargeted Metabolite Analysis Reveals Distinct Responses between Communities

To gain a broader perspective of how CAP impacts the production of metabolites by the gut microbiota, an untargeted metabolomics analysis was combined with a regression analysis to correlate bacterial species with the metabolites that were produced by the microbial communities. Since this was an untargeted analysis, this is a correlation that these products were likely to be present within these particular bacteria. Many of the compounds identified were identified as being “similar to”, regarding structural similarity.

**Figure 5:**
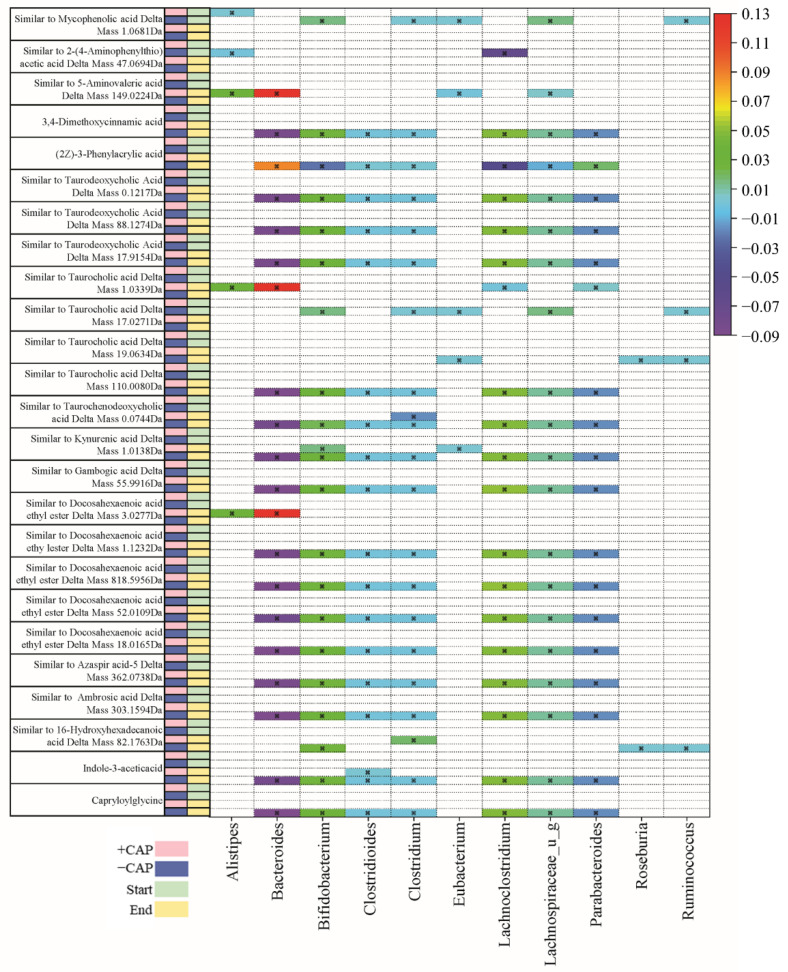
Table of C1 community correlations of metabolites to key bacterial species, from CAP Start to CAP End. Significant correlations were determined by *q* < 0.05.

**Figure 6:**
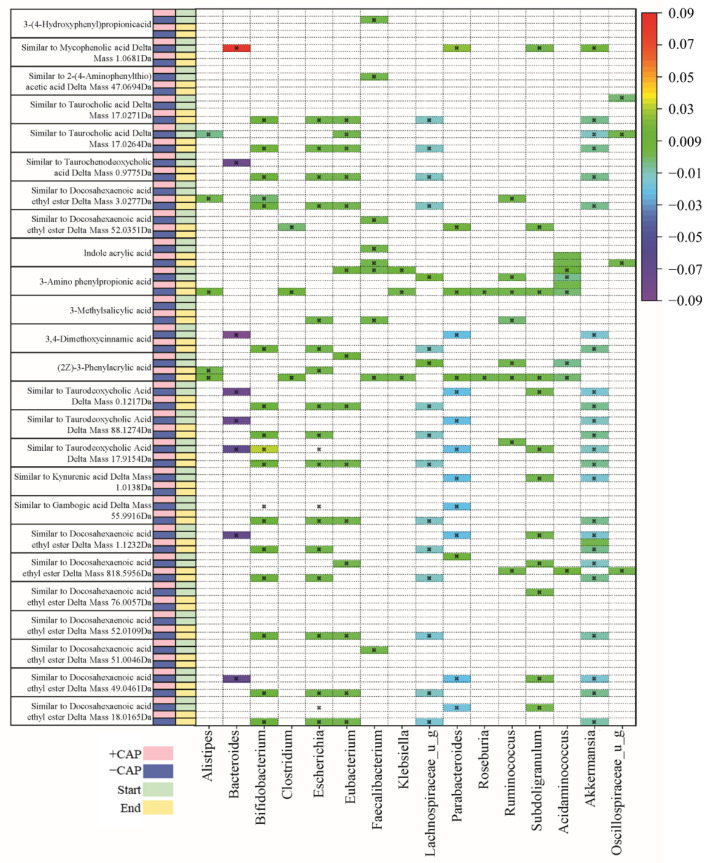
Table of C2 community correlations of metabolites to key bacterial species, from CAP Start to CAP End. Significant correlations were determined by *q* < 0.05.

Two heat maps that summarize these results can be found in Figure 5 and Figure 6. There was little overlap in the significant correlation (*q* < 0.05) of metabolites to bacteria between C1 (Figure 5) and C2 (Figure 6). Data were filtered by the bacteria of interest, identified by previous studies on CAP and changes in the gut microbiota in this particular study, depending on whether it was C1 or C2 [24,26,28]. Potential metabolites in the heatmap are those that exhibited a significant correlation coefficient with a particular genus. 

The majority of metabolites detected were fatty acids and bile acids. Docosahexaenoic acid ethyl ester is a long-chain fatty acid (Figure 5) and it has previously been positively correlated with *Bacteroides* [55]. In this study, we found multiple instances of compounds similar to docosahexaenoic acid ethyl ester that were negatively associated with *Bacteroides* and *Parabacteroides* in C1 –CAP (control) but positively correlated with *Bacteroides* and *Alistipes* in C1 +CAP (*q* < 0.05) (Figure 5), particularly at the CAP End. At the CAP End in the C2 community, compounds similar to docosahexaenoic acid ethyl ester had a positive correlation with *Bifidobacterium* but a negative correlation with *Escherichia* and *Lachnospiraceae*. No significant correlations were found with the +CAP communities in C2. 

Kynurenic acid is another metabolite prevalent across genera, which is produced by the metabolism of L-tryptophan [56]. It has been demonstrated to have positive effects on gastrointestinal health in terms of colonic disease [57]. In this study, a compound similar to kynurenic acid had a significantly positive correlation in C1 –CAP at the CAP End with *Lachnospiraceae, Lachnoclostridium,* and *Bifidobacterium* (*q* < 0.05) but no significant correlation with the CAP treatment in either C1 or C2, which indicates that Kynurenic acid has no significant relationship with CAP use. 

Taurocholic acid is a conjugated bile acid made from cholic acid and taurine whose absorption levels from the gut are lowered in cystic fibrosis models [58,59]. In this study, compounds found that are similar to taurocholic acid were significantly correlated with all listed genera in C1 (Figure 5); however, they were found in the –CAP community (*q* < 0.05). In C2 (Figure 6), these compounds similar to taurocholic acid were associated with *Alistipes* and *Lachnospiraceae*, exhibiting a negative correlation, and a positive correlation with *Eubacteria,* again only in the –CAP community (*q* < 0.05). Compounds similar to taurodeoxycholic acid, which is another bile acid and conjugate of deoxycholic acid, were also associated with most listed genera, including *Bacteroides*, *Bifidobacterium*, *Chlostridioides*, *Lachnospiraceae*, and *Parabacteroides*. Phenylacrylic acid (cinnamic acid) was correlated with *Bacteroides*, *Bacteroidales*, *Acidaminococcus*, *Alistipes*, *Parabacteroides*, *Ruminococcus*, and *Roseburia* (*q* < 0.05). Taurochenodeoxycholic acid is another conjugated bile acid, formed from chenodeoxycholic acid and taurine. This bile acid was found to be associated with *Bacteroides*, *Escherichia*, *Lachnospiraceae*, and *Parabacteroides* (*q* < 0.05). The fatty acid aminovaleric acid was found to be correlated with *Bacteroidales* and *Alistipes* (*q* < 0.05)*. Bacteroidales*, *Acidaminococcus*, and *Alistipes* were correlated with 3-Amino phenylpropionic acid in the C2 –CAP group (*q* < 0.05). Furthermore, 3,4-Dimethoxycinnamic acid was associated with *Bifidobacterium*, *Escherichia*, and *Lachnospiraceae* (*q* < 0.05). 

In the *Bacteroides* genus, metabolites were found to have a slight negative correlation in the C1 –CAP group, whereas those correlations that were significant had a slight positive correlation in the C1 +CAP group. Most of the metabolites found were similar to bile acids and fatty acids that are common within gut microbial communities. Most significantly correlated metabolites were found in the control groups of both C1 and C2. This may indicate that CAP reduces the bacteria that are correlated with the presence of certain metabolites or that the metabolites were undetectable in the CAP samples. Most of the metabolites discovered using this method of untargeted metabolomics were bile acid conjugates or a type of fatty acid, compounds typically found in a functioning gut microbial community. 

Overall, when the diversity measures (Figure 1 and Figure 2), proportional abundance data (Figure 3), SCFA analysis (Figure 4), and untargeted metabolomics data (Figure 5 and Figure 6) are taken together, it is clear that the C1 and C2 bacterial communities responded strongly to CAP, and that this response differed depending on the community composition. 

## 4. Conclusions

Historically, CAP was used for digestive issues and pain management, and more recent studies have worked to find the mechanisms for those health benefits [1,2]. More recent work has established CAP as a beneficial addition to the diet to help protect against high cholesterol and obesity and to improve glucose homeostasis, although the longevity of these effects and which dose is best are still unknown [9,24,26,27]. These same beneficial health effects are often seen through the consumption of other food compounds, such as dietary fiber and polyphenols [60,61]. The current study is in agreement with previous work that CAP alters the gut microbial community structure by increasing the diversity of the community. This study also illustrated that CAP can shift SCFA abundance, which is a potential explanation for its beneficial health effects [8,9]. We observed these shifts in abundance through the increase of propanoic acid shown in C1 and C2, as well an increase of butanoic acid abundance found in C1. A similar increase in propanoic acid and butanoic acid has been found in vitro in response to dietary fiber from sweet potatoes [33]. Untargeted metabolomics, however, revealed that CAP exposure reduced the number of significant associations between particular bacteria and certain metabolites when compared with an untreated control. This change is likely due to the observed shifts in the gut microbial structure. 

While performing an in vitro study does remove the host component from our analysis and therefore is limited in scope, our study design has several advantages. The removal of the host tissues allows the effects of the compound on the bacterial community alone to be discovered. It is also advantageous to perform metabolomics and the SCFA analysis from a culture microbiome, as analyzing from fecal samples alone will illustrate what compounds last until collection. This work identified multiple microbial changes as a function of CAP that may prove beneficial to host physiology. Overall, our results found that CAP significantly altered the gut microbial structure and SCFA levels; however, these changes were donor-dependent.

## Data Availability

The data is this study is available in the Supplementary Materials.

## Supplementary Materials

The following are available online at https://www.mdpi.com/article/10.3390/nu14061283/s1, Table S1: SCFA data, Table S2: Untargeted Metabolomics Using LC-MS C1 (*q* < 0.05), Table S3: Untargeted Metabolomics Using LC-MS C2 (*q* < 0.05), Table S4: OTU data.
